# Effect of *Bacillus subtilis* on mechanical and self-healing properties in mortar with different crack widths and curing conditions

**DOI:** 10.1038/s41598-023-34837-x

**Published:** 2023-05-15

**Authors:** Nattapong Yamasamit, Panisa Sangkeaw, Wittaya Jitchaijaroen, Chanachai Thongchom, Suraparb Keawsawasvong, Viroon Kamchoom

**Affiliations:** 1 Department of Civil Engineering, Faculty of Engineering, Thammasat School of Engineering, Thammasat University, Pathum Thani, Thailand; 2 Excellent Centre for Green and Sustainable Infrastructure, Department of Civil Engineering, School of Engineering, King Mongkut’s Institute of Technology Ladkrabang (KMITL), Bangkok, Thailand

**Keywords:** Engineering, Civil engineering

## Abstract

This research aimed to investigate the effectiveness of *Bacillus subtilis* (*B. subtilis*) in self-healing cracks in concrete and enhancing concrete strength through microbial induced calcium carbonate precipitation (MICP). The study evaluated the ability of the mortar to cover cracks within 28 days, taking into account the width of the crack, and observed the recovery of strength after self-healing. The use of microencapsulated endospores of *B. subtilis* was also examined for its impact on the strength of concrete. The compressive, splitting tensile, and flexural strengths of normal mortar were compared to those of biological mortar, and it was found that biological mortar had a higher strength capacity. Microstructure analysis using SEM and EDS showed that bacterial growth increased calcium production, contributing to the improved mechanical properties of the bio-mortar.

## Introduction

The durability and lifespan of concrete structures can be negatively impacted by the formation of cracks over time, either due to internal volume instability or external stress factors such as heavy loads. These cracks can pose a threat to the integrity of the main structure and are often caused by natural events such as earthquakes, weathering, or human activities. It is crucial to address these cracks in order to maintain the stability and longevity of the concrete structures^1^. The production of cement is a major contributor to carbon dioxide emissions, which can lead to the greenhouse effect, climate change, and global warming. It is a well-known fact that the cement industry is a significant source of greenhouse gas emissions, and efforts to reduce these emissions are becoming increasingly important for the preservation of our planet's health and future^2,3^.

Various techniques have been developed to address the problem of cracks in concrete, including autogenous healing, polymer injection, and microbial self-healing. Autogenous healing is an inherent process that can take place in concrete as a result of the hydration of unreacted cement particles with water, leading to the formation of calcium silicate hydrate that can seal cracks. Polymer injection involves injecting a polymer resin into the crack, which then expands to fill the crack^4^. In contrast, microbial self-healing employs microorganisms, such as bacteria to repair cracks in the concrete by microbially induced carbonate precipitation (MICP), the MICP process requires a urea (substrate) by urea hydrolysis process using urease enzymes^5,6^, it produces by microbial to disintegrate into CO_3_^2−^ and NH_4_^+^ and through biochemical activity can form calcium carbonate precipitation (CaCO_3_) as the Eqs. (1) and (2) ^7–9^.

1 $$\text{CO(N}{\text{H}}_{2}{\text{)}}_{2}+ \text{2} {\text{H}}_{2}\text{O }\stackrel{\text{Urease}}{\to } \, {\text{2N}}{\text{H}}^{4+}+ \text{ } {{\text{CO}}_{3}}^{2-},$$

2 $${\text{Ca}}^{2+}+ \text{ } {{\text{CO}}_{3}}^{2-} \, \stackrel \, {\to } \, {\text{CaCO}}_{3}.$$

In process MICP does not produce carbon dioxide during the calcium carbonate precipitation. MICP is a green environmental protection and widely used in the field environmental and civil engineering such as the bio cement and healing crack concrete by MICP^10^. The microbial is commonly used to study the MICP is bacteria in *Bacillus* species, it was gram positive bacteria and could form endospore because when the bacteria were resistant condition to high pH (pH 12–13) of concrete and inappropriate environment^6,11–17^.

In this study, *Bacillus subtilis* (*B. subtilis*) was selected as the microbial agent of focus due to its environmental safety and lack of adverse physiological effects on humans (bacteria risk group 1). Mixing bacteria with cement mortar can expose the bacteria to harsh conditions, including high pH levels, which can lead to cell damage and death^18^. Encapsulating the bacteria in a protective layer can mitigate such damage by providing a physical barrier between the bacteria and the cement mortar. The encapsulation layer can also regulate the diffusion of chemicals and other harmful substances, which helps maintain a stable microenvironment around the encapsulated bacteria^13,18^. This research focused on the characterization of *B. subtilis* as a microbial agent for microencapsulation and controlled release (MICP) in Thailand, where it was isolated from soil and identified using conventional methods and biochemical testing. *B. subtilis* was chosen for its ability to produce the urease enzyme for urea hydrolysis, its resistance to environmental stressors, and its non-pathogenic nature for human health^19^.

The aim of this study was to evaluate the efficacy of *B. subtilis* in healing crack width through the application of vegetative cells on the mortar surface and incorporating *B. subtilis* fragments into the mortar mixture. The performance of *B. subtilis* in the microencapsulation and controlled release process (MICP) for crack healing is monitored through the use of a stereo microscope, as well as tests for water absorption and mechanical properties. Microstructural analysis is also performed using scanning electron microscopy (SEM) and energy dispersive X-ray spectroscopy (EDS) to further understand the mechanisms of crack healing in the bio mortar.

## Material

### Material cement and fine aggregate

Cement is mainly a binding medium which is hardened with the other materials in a mortar producing a homogeneous mass. Cement that we have used in this research is Ordinary Portland Cement (OPC Type I). Fine aggregate for fill the voids in mortar that we have used in this research is River Sand. A sample of 500 g was tested for water absorption and the result is 1%^20^.

### Bacteria and microencapsulation

#### Bacteria for self-healing

**Figure 1:**
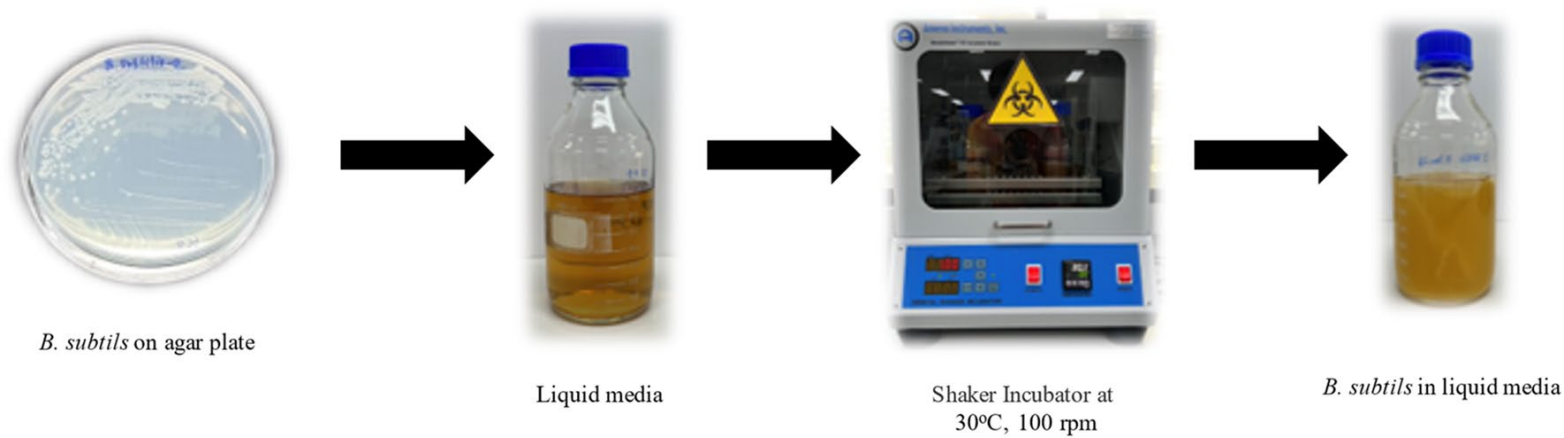
Process of prepared bacteria for self-healing.

The study used *B. subtilis* it isolated from soils in Thailand by conventional method and confirmed species of bacteria by biochemical test. Growth the *B. subtilis* on the Tryptic soy agar (TSA) and incubate at 30 °C, 24 h. Therefrom, transfer the *B. subtilis* on TSA plate to the liquid medium (Tryptic soy broth (TSB) 40 g/l, NaHCO_3_ 2.12 g/l, and Urea 10 g/l) before use the media was autoclave at 121 °C for 15 min. The cultures were incubated at 30 °C, 100 rpm for 24 h and diluted the cell in media to 10^8^ CFU/ml while prepared Urea—CaCl_2_ broth (Urea 0.25 molar and CaCl_2_ 0.375 molar adjust pH = 8 by NaHCO_3_). Finally mix *B. subtilis* in Urea—CaCl_2_ broth for drop on crack mortar ^21^. Figure 1 presents the process of prepared bacteria for self-healing.

#### Microencapsulation

**Figure 2:**
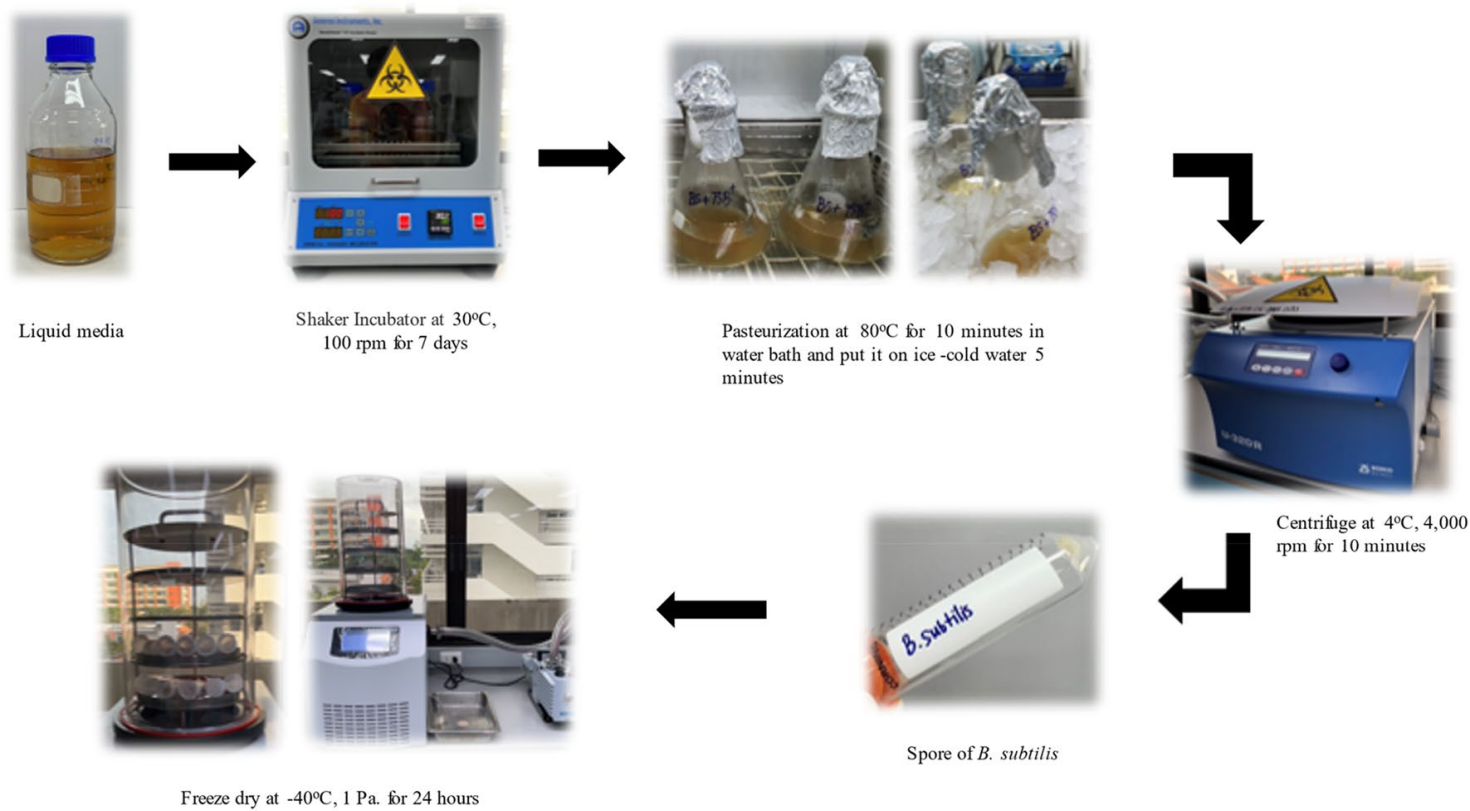
Process of microencapsulation.

Microencapsulation is a process used to protect and preserve the viability of the bacterial spores in this research. To grow B. *subtilis*, it was first cultured on Tryptic Soy Agar (TSA) at 30 °C for 24 h. The cultured bacteria were then transferred to a liquid media consisting of Tryptic Soy Broth (TSB), NaHCO_3_, and Urea that was autoclaved at 121 °C for 15 min. The cultures were incubated at 30 °C and 100 rpm for 7 days. After pasteurizing the liquid media at 80 °C for 10 min, it was put on ice-cold water for 5 min to remove the vegetative cells. The spores were separated from the liquid media by centrifugation at 4 °C, 4000 rpm for 10 min. The spores were washed with 1% sodium chloride-peptone water and diluted to a concentration of 10^8^ cell/mL. The spores were then encapsulated in a 2% sodium alginate solution, frozen at − 80 °C for 24 h, and subjected to freeze-drying techniques at a condenser temperature of − 40 °C and a chamber pressure of 1 Pa^13,22^. The procedure is shown in Fig. 2.

### Specimen details

The test specimens for different of tests used in this research. Cube specimens of 50 × 50 × 50 mm. is used for compressive strength test, compressive strength recovery test and water absorption test. Beam specimens of 40 × 40 × 160 mm. is used for flexural strength test. Cylinder specimens of 50 × 100 mm diameter is used for splitting tensile strength test.

## Methodology

### Microcapsules specimens

**Figure 3:**
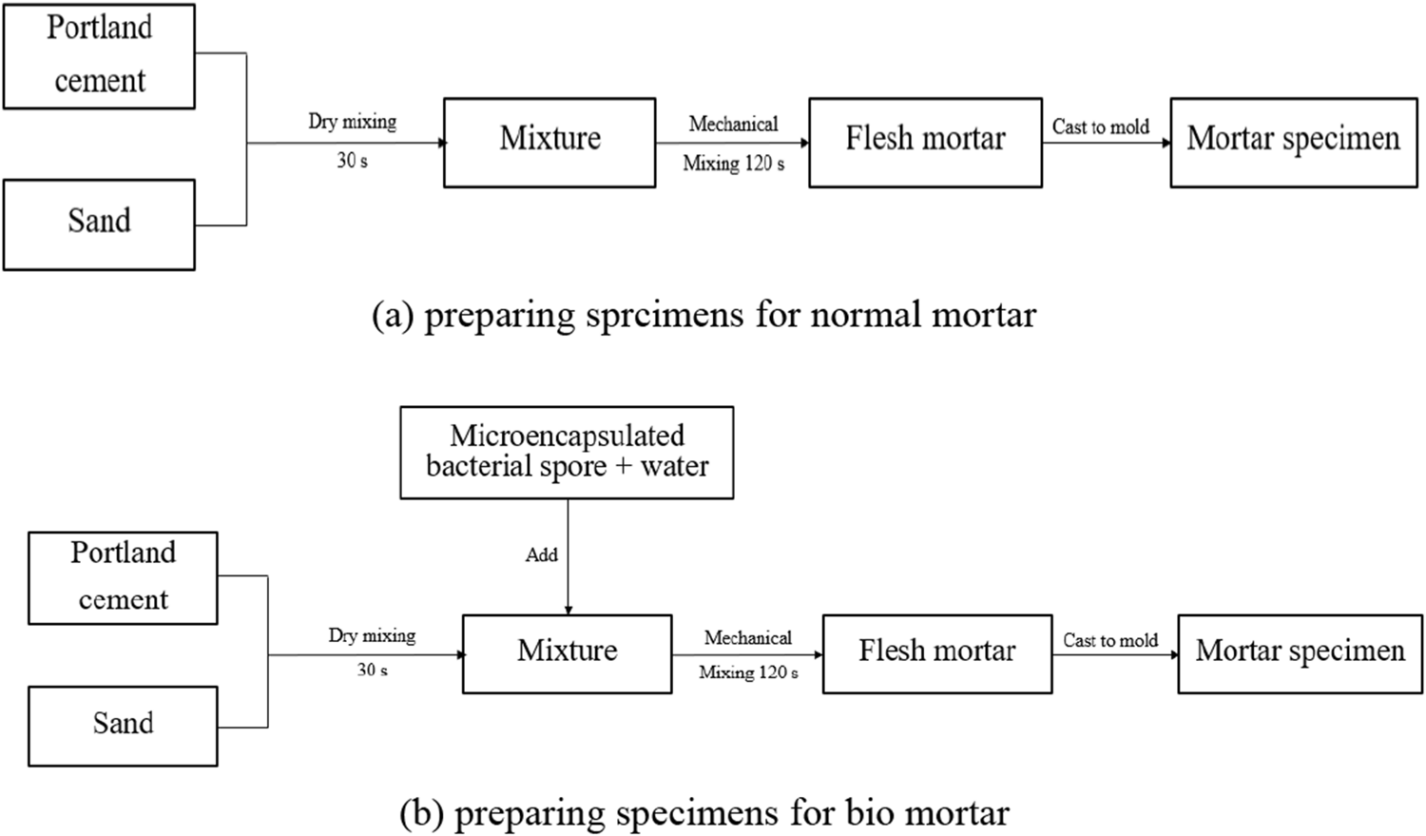
Procedure for mixing normal and bio mortars.

In this research, mortar specimens were prepared with a water to cement ratio (w/c) of 0.5 and sand to cement ratio (s/c) of 2.75. The procedure for preparing bio mortar involves first preparing tap water and then using microcapsules, which are cross-linked using a 2% (w/v) CaCl_2_ solution for 30 min^6^. The microcapsules are then added to water at a rate of 1.8% (w/w) dry cement. This mixture is used to cast the bio mortar. Table 1 presents the concrete mixture proportions for the normal and bio mortar. And, the mixing procedures for the normal and bio mortar specimens are presented and Fig. 3.

**Table 1:** Concrete mixture proportions.

Specimens	Cement (g)	Water (g)	Sand (g)	Microencapsulated bacterial spore (g)
Normal mortar	100	50	275	–
Bio mortar	100	50	275	1.8

### MICP specimens (self-healing)

In this research, the test mortar specimens for MICP had a water-to-cement-to-sand ratio of 1:2:5. Cube specimens of 50 × 50 × 50 mm were used to simulate outside crack testing. Cracks were made on the specimens with a copper sheet of 0.3, 0.5, and 1.0 mm size and then cured in water for 28 days. The width of a crack is a key parameter that can provide important insights into the severity of damage to a concrete structure. Cracks with a width of 0.3 mm or less are typically categorized as hairline cracks, which are superficial and do not pose a significant threat to the structural integrity of the concrete. In contrast, cracks with a width of 0.5 mm are generally classified as medium cracks, which can indicate that the structure is undergoing significant stress and movement. If left untreated, medium cracks can compromise the durability and long-term stability of the structure. Finally, cracks with a width of 1.0 mm or greater are commonly regarded as wide cracks, which can signify significant movement or excessive loading that require immediate attention to prevent further damage to the structure.

**Figure 4:**
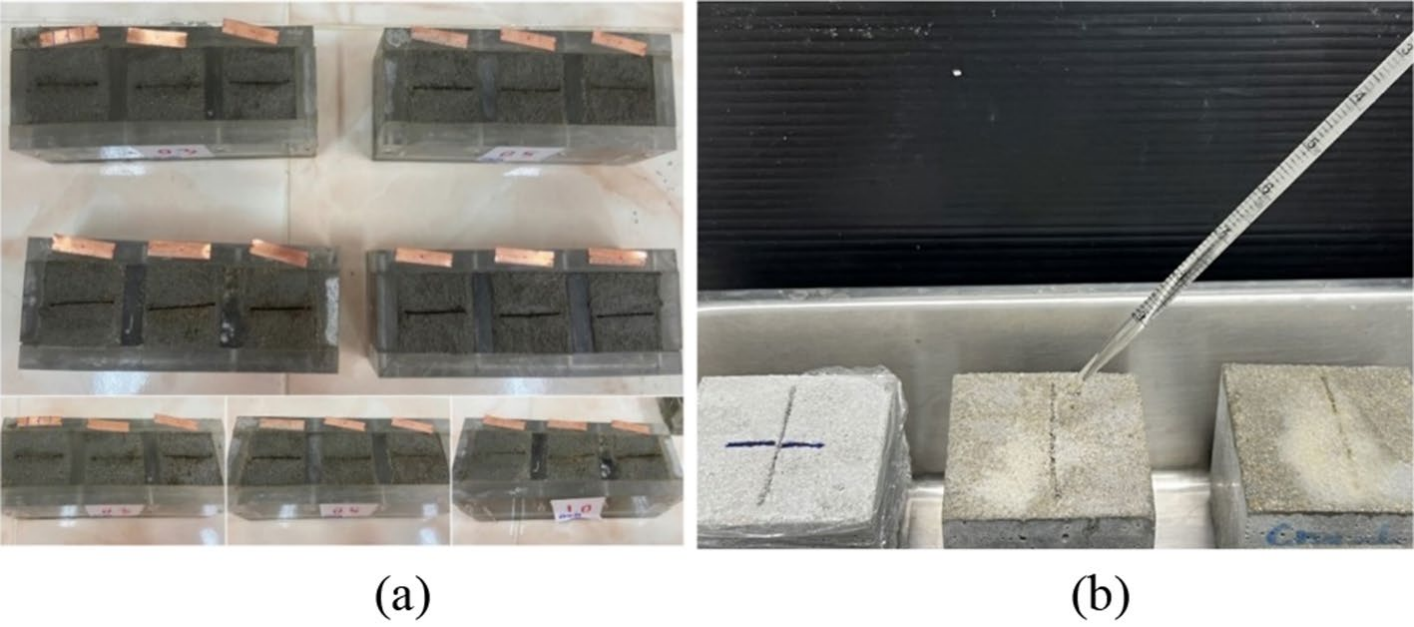
(**a**) Creation of cracks with 0.3, 0.5 and 1.0 mm copper plates. (**b**) Dropping solution culture for healing crack.

This study aimed to investigate the effectiveness of different healing agents on the crack healing of mortar specimens. Generally, two common methods for filling cracks are spraying and dropping. In the sprayed method, the healing agent is sprayed onto the surface of the cracked mortar, while in the dropping method, the healing agent is dropped directly onto the surface of the crack. In this study, the dropping method was chosen as the preferred method for healing the cracks on the surface of the mortar specimens. This method involves dropping the healing agent directly onto the surface of the cracked mortar. It was chosen over the sprayed method as it was deemed more effective for the specific type of cracks that were present in the specimens being tested. To test the efficiency of the healing process, *B. subtilis* was dropped into CaCl_2_ broth and urea solution on the crack surface of the specimens every 24 h and incubated at a temperature of 30 °C for 7, 14, and 28 days, as shown in Fig. 4. The size of the cracks was measured daily to monitor the healing progress by crack width comparator gauge for measuring the crack width and analyze of crack width by microscope. The healing ratio for evaluate performance was calculated using the following equation.

3 $$\text{Healing ratio }(\text{\%}) = \frac{\left|\text{After crack width}-\text{Initial crack width}\right|}{\text{Initial crack width}}\times 100$$

### Absorption properties

The water absorption of the specimens was assessed by observing changes in their weight after soaking and measuring the surface absorption according to ASTM C1585^23^. The water absorption was calculated using the following equation.

4 $${\text{Absorption}} (\% ){ = }\frac{{{\text{W}}_{{\text{s}}} - {\text{W}}_{{\text{d}}} }}{{{\text{W}}_{{\text{d}}} }} \times 100.$$

### Mechanical properties

The compressive strength, splitting tensile strength, and flexural strength tests were conducted with a loading rate of 0.5 mm/min until failure.

The compressive strength (f_a_) was determined using the standard ASTM C109^24^ method and was calculated from the following equation.

5 $${\text{f}}_{{\text{a}}} = \frac{{\text{P}}}{{\text{A}}} \times 10,$$

where, P is ultimate load of specimens and A is cross-sectional area.

The splitting tensile strength (f_t_) was determined using the standard ASTM C496^25^ and was calculated using the following equation.

6 $${\text{f}}_{{\text{t}}} = \frac{{{\text{2P}}}}{{\pi {\text{LD}}}} \times 10,$$

where, L is Length of specimens, and D is diameter of specimens.

The flexural strength test was conducted using the standard ASTM C78^26^, and the results were calculated using the following equation.

7 $${\text{R}} = \frac{{{\text{3LP}}}}{{2bd^{2} }} \times 10,$$

where, b and d are width and depth of specimens respectively.

## Result and discussion

### Water absorption

**Figure 5:**
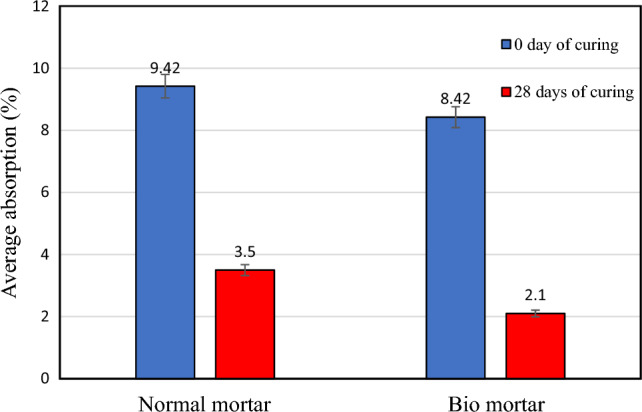
Average absorption of normal and bio mortars.

The addition of microcapsules resulted in a reduction of water absorption for the specimens, as shown in Table 2 and Fig. 5. The average water absorption for normal mortar was 9.42% at 0 days and 3.5% at 28 days, while the average water absorption for bio mortar was 8.42% at 0 days and 2.1% at 28 days. Normal mortar was found to have higher water absorption than bio mortar because of the MICP process precipitates calcium carbonate, it fills the microcracks in the natural porous structure of the mortar and reduces its porosity^27^.

**Table 2:** Water absorption of normal and bio mortar.

Specimens	Curing age (days)	Average absorption (%)
Normal mortar	0	9.42
	28	3.50
Bio mortar	0	8.42
	28	2.10

### Compressive strength

The results of compressive strength of specimen (normal and bio mortar) at 7 and 28 days, in water curing and plastic curing as shown in Table 3.

**Table 3:** Compressive strength (MPa) of the specimen.

Curing	Normal mortar		Bio mortar	
	7 days	28 days	7 days	28 days
Water	35.6	45.1	37.6	49.1
Plastic	34.3	36.6	36.6	45.9

**Figure 6:**
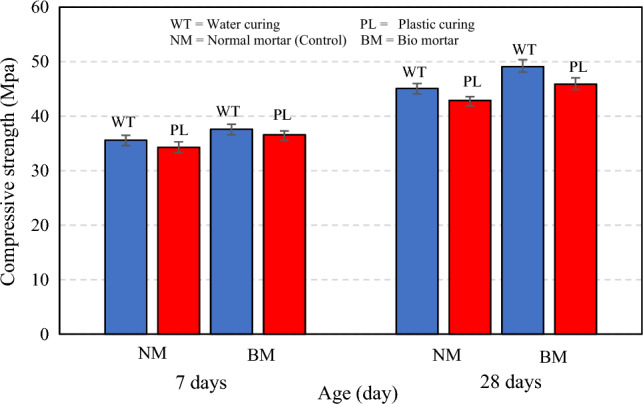
Compressive strength of normal and bio mortars.

The addition of microcapsules had a positive effect on the mechanical properties of the specimens (water and plastic curing) compared with that of mixture control. As shown in Fig. 6 the compressive strength at 7 days of normal and bio mortar in water curing was 35.6 and 37.6 MPa, in plastic curing was 34.3 and 36.6 MPa, respectively. The compressive strength was increased by 5.6% in water curing and 6.7% in plastic curing. The compressive strength at 28 days of normal and bio mortar in water curing was 45.1 and 49.1 MPa, in plastic curing was 42.9 and 45.9 MPa. The compressive strength increased by 8.9% in water curing and 7% in plastic curing. However, test results when compare two methods of curing it was found that in normal mortar curing with water curing the compressive strength at 28 days was 23.2% higher than plastic curing. For bio mortar curing with water curing the compressive strength at 28 days was 7% higher than plastic curing.

### Splitting tensile strength and flexural strength

The results of splitting tensile strength and flexural strength of specimen (normal and bio mortar) at 7 and 28 days, in water curing and plastic curing as shown in Tables 4 and 5.

**Table 4:** Splitting tensile strength (MPa) of the specimens.

Curing	Normal mortar		Bio mortar	
	7 days	28 days	7 days	28 days
Water	2.7	3.0	2.9	3.4
Plastic	2.7	3.0	2.9	3.2

**Table 5:** Flexural strength (MPa) of the specimens.

Curing	Normal mortar		Bio mortar	
	7 days	28 days	7 days	28 days
Water	6.0	8.6	6.1	9.2
Plastic	6.0	8.6	6.1	9.0

**Figure 7:**
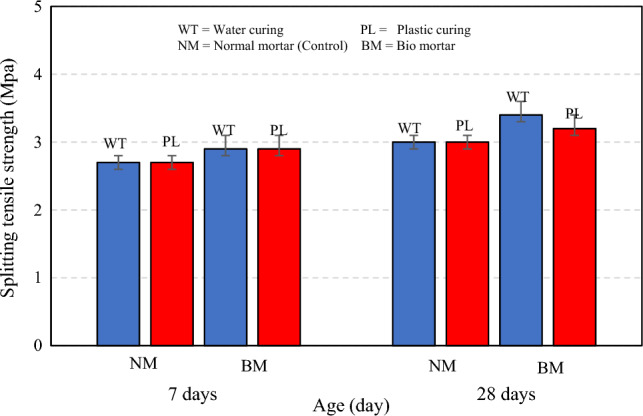
Splitting tensile strength of normal and bio mortars.

As shown in Fig. 7, the splitting tensile strength at 7 days of normal mortar in water and plastic curing was 2.7 MPa, and bio mortar in water and plastic curing was 2.9 MPa, respectively. The splitting tensile strength was increased by 7.4% in water and plastic curing. The splitting tensile strength at 28 days of normal mortar in water and plastic curing was 3.0 MPa and bio mortar in water curing was 3.4 MPa, and in plastic curing was 3.2 MPa. The splitting tensile strength was increased by 13.33% in water curing and 7.4% in plastic curing. However, test results when compared two curing methods, it was found that in normal mortar curing with water and plastic were equal. For bio mortar curing with water the splitting tensile strength at 28 days was 6.3% higher than plastic curing.

**Figure 8:**
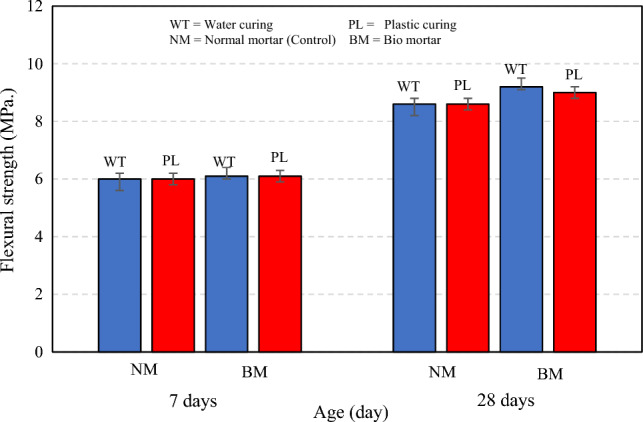
Flexural strength of normal and bio mortars.

The flexural strength at 7 days of normal and bio mortar no difference was observed of mixture control. At 28 days of normal in water and plastic curing was 8.6 MPa and bio mortar in water curing was 9.2 MPa, and in plastic curing was 9.0 MPa. The flexural strength increased by 7% in water curing and 4.7% in plastic curing. However, test results when compare two methods of curing it was found that in normal mortar curing with water and plastic were equal. For bio mortar curing with water curing the flexural strength at 28 days was 2.2% higher than plastic curing, as shown in Fig. 8.

### Self-healing efficiency

**Figure 9:**
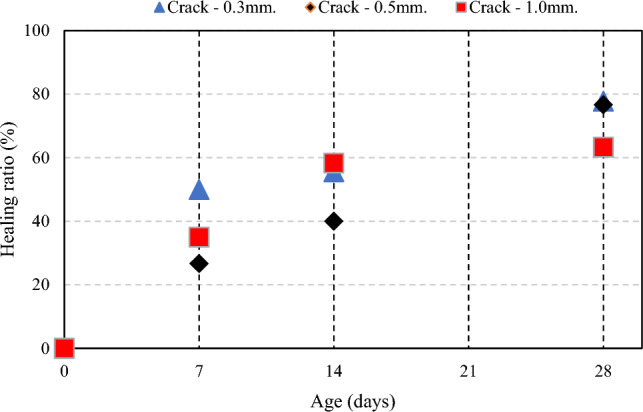
Healing ratio in specimens under different crack width.

**Figure 10:**
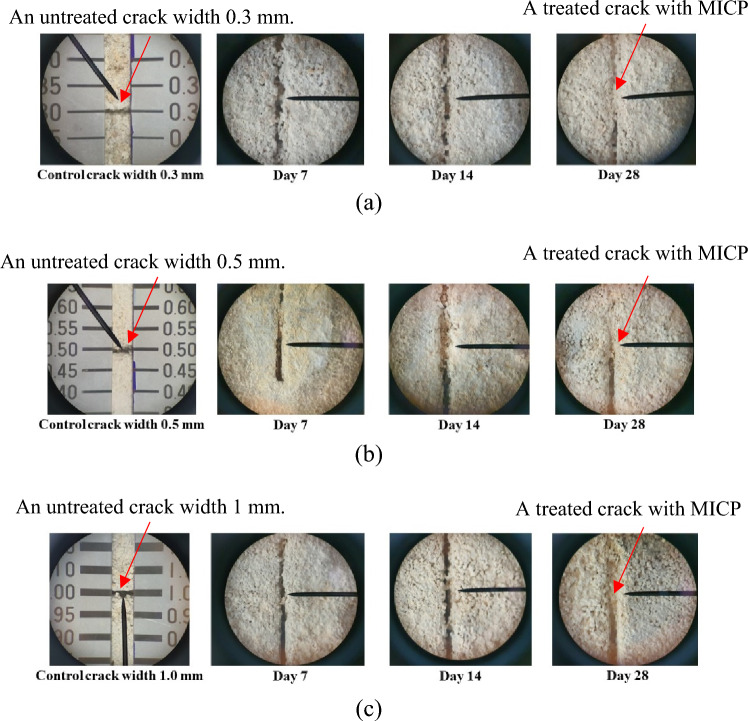
Surface area cracking of specimens mortar and days of healing crack (**a**) crack width 0.3 mm (**b**) crack width 0.5 mm (**c**) crack width 1.0 mm.

Test results show efficiency of crack healing was observed in all specimens. The specimens with treatment by MICP, dropping the CaCl_2_ broth with *B. subtilis* and urea solution at T = 30 °C. The average crack width in the specimens equal to 0.3 mm. had a healing ratio of 50%, 55.56% and 77.78% at 7, 14 and 28 days (age of mortar). The average crack width in the specimens equal to 0.5 mm. had a healing ratio of 26.67%, 40% and 76.67% at 7, 14 and 28 days. For the average maximum crack width in the specimens equal to 1 mm. had healing ration of 35%, 58.33% and 63.33% at 7, 14 and 28 days because the healing process, the crack are filled with visible white mineral calcium carbonate. *B. subtilis* can form CaCO_3_ through the urea hydrolysis course under low nutrient conditions. In addition, calcite, aragonite, C–S–H and ettringite with more repaired and healed^28,29^, as shown in Fig. 9. The images of the difference width crack in specimens with self-healing difference healing stage. It can be considered that crack was healing by white precipitations. The crack width of 0.3, 0.5 and 1 mm. was almost full healing at 28 days and perform of analyze at crack surface by microscopic analysis of the indicated precipitation, as shown in Fig. 10.

### Compressive strength recovery for self-healing mortar

**Figure 11:**
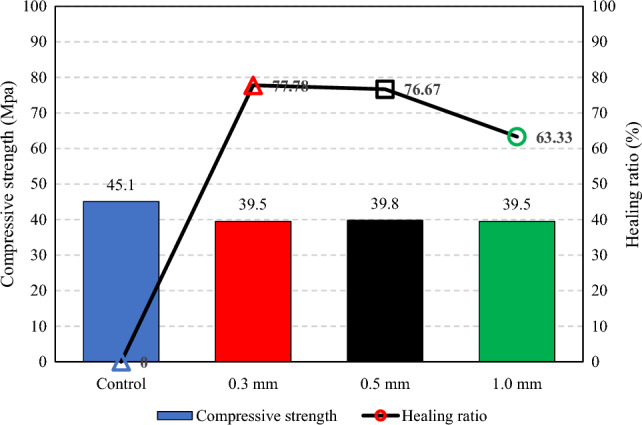
Relationship between compressive strength recovery and healing ratio for self-healing mortar at 28 days.

After the mortar specimens treatment by MICP, dropping the CaCl_2_ broth with *B. subtilis* culture and urea solution every 24 h. at temperature at T = 30 °C and after for 28 days, was carried out to determine the compressive strength recovery rate due to microbial self-healing. After 28 days of healing for the specimens had crack width 0.3, 0.5 and 1 mm. had the recovery strength of mortar was 39.5, 39.8 and 39.5 MPa. The compressive strength decreased by 14.2%, 13.32% and 14.2% and relationship between compressive strength recovery and healing ratio for self-healing mortar at 28 days, as shown in Fig. 11.

### Microstructural analysis

**Figure 12:**
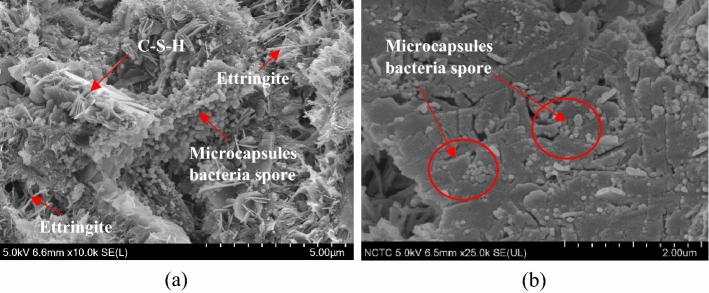
Surface of bio mortar specimen. (**a**) Microcapsule in CSH gel and ettringite matrix. (**b**) Microcapsule in matrix.

Microstructural analysis techniques, including SEM, elemental mapping, and qualitative analysis, were used to examine the specimens. SEM analysis can be used to evaluate the bonding between the microcapsules and mortar matrix. The microstructure of the mortar was observed to change due to the addition of microencapsulated bacterial spores. In our study, SEM images were analyzed to compare the bonding between the bio mortar with microencapsulated 1.8 g and normal mortar. As shown in Fig. 12, each microcapsule was found to meet the required processing standards and was not damaged due to the mortar mixing procedure. The microcapsules showed good bonding with the matrix in mortar, as evidenced by the presence of capsule shells remaining at the surface of the matrix. In addition, the microcapsules were embedded within the matrix structure (as shown in Fig. 12b), indicating that they contributed to the decrease in pore size within the matrix. This corresponds to the observed increase in mechanical properties and decrease in water absorption in the bio mortar. Elemental mapping analysis also confirmed the presence of the encapsulated bacterial spores in the mortar matrix, further indicating their good bonding.

**Figure 13:**
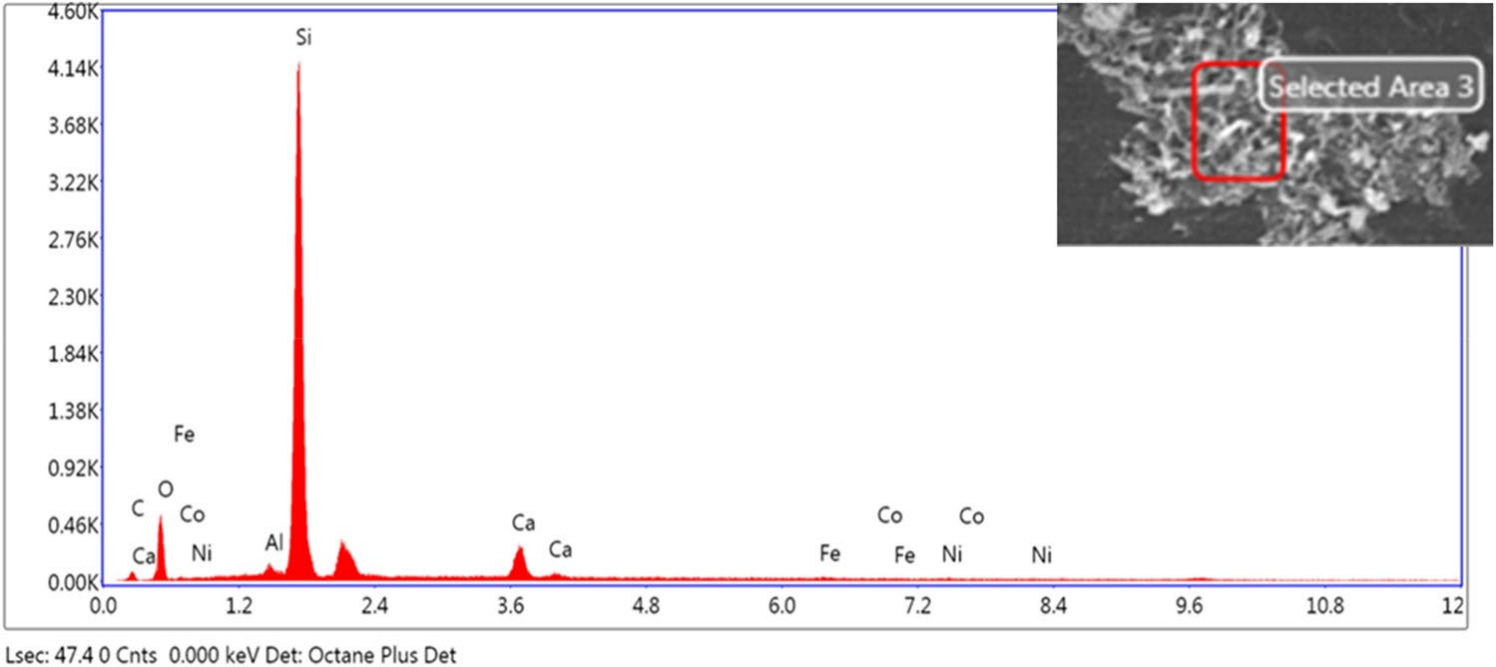
EDS elemental analysis for a crack surface in specimens.

**Figure 14:**
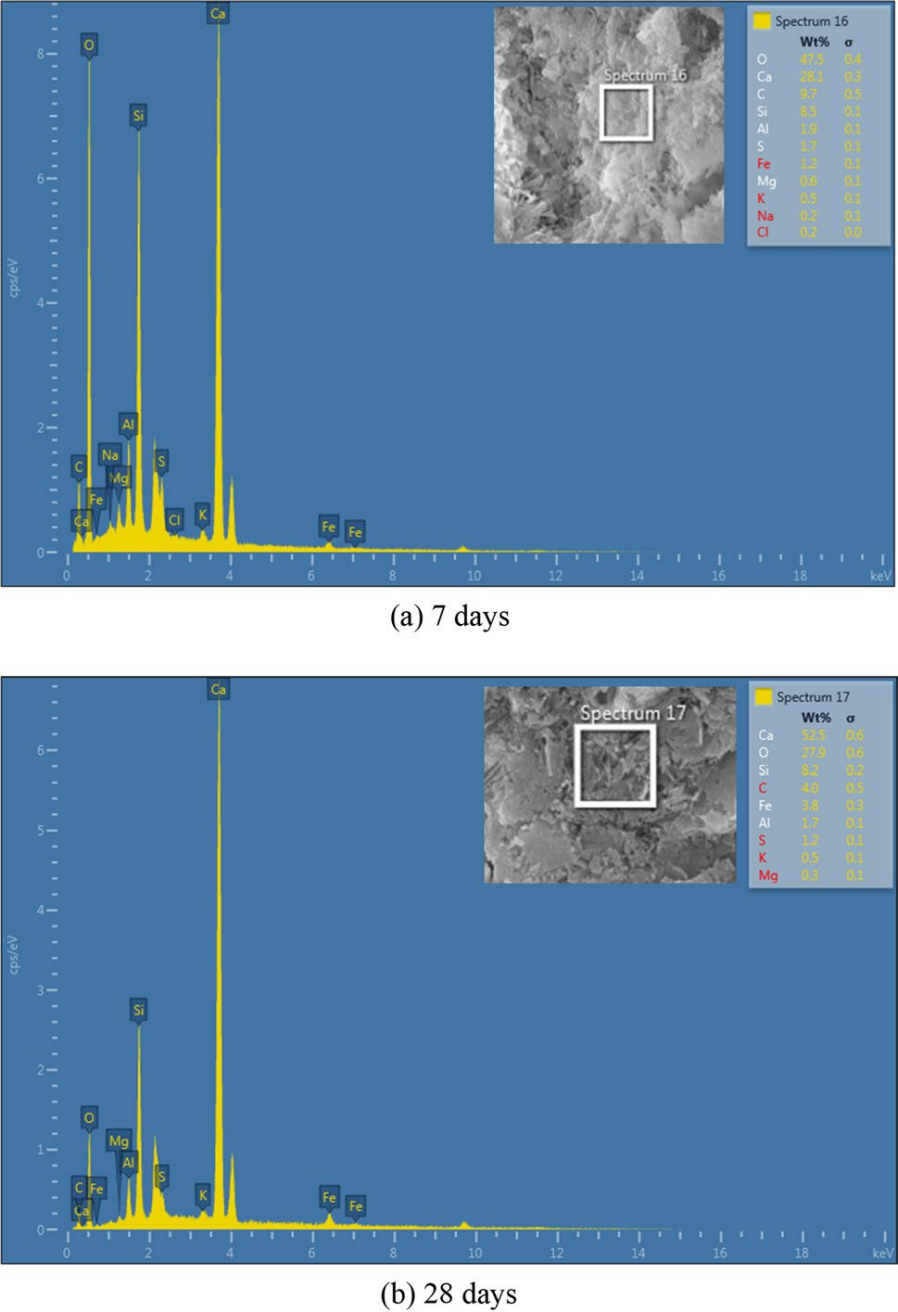
Element composition of bio mortar.

In addition to microstructure of bio mortar in this study have to analy the composition of elements in bio mortar for %weight of elements had change for 1 (shown in Fig. 13 and Table 6), 7 and 28 days (shown in Fig. 14a,b) by energy dispersive X-ray spectroscopy (EDS). The EDS has shown the compose of elements Calcium (Ca), Carbon (C), and Oxygen (O) in the bio mortar. The composition of element Ca in bio mortar at 1, 7, and 28 days increased by 8.73, 28.1 and 52.5 weight %, element C in the specimen at 1, 7, and 28 days lessened by 12.77, 9.7, 4.0 weight %. In process of MICP have factor for bacterial growth in us to produce enzyme urease for urea hydrolysis, substance in precipitation calcium carbonate therefor the zone has composition of element carbon (C), calcium (Ca), and oxygen (O).

**Table 6:** Energy dispersive X-ray spectroscopy (EDS).

Element	Weight %	Atomic %	Error %
C	12.77	22.89	15.47
O	22.70	30.54	10.67
Al	0.04	0.03	68.63
Si	53.50	41.00	2.77
Ca	8.73	4.69	7.42
Fe	0.92	0.35	59.60
Co	0.35	0.13	61.93
Ni	1.00	0.36	58.58

## Conclusions

Based on the experimental investigation, the following conclusions can be drawn:

- The use of microencapsulated *B. subtilis* has the potential to improve the mechanical and absorption properties of mortar. Compared to normal mortar, the use of *B. subtilis* resulted in an increase of 7–8.9% in compressive strength, an increase of 7.4–13.33% in splitting tensile strength, an increase of 4.7–7% in flexural strength, and a decrease of 75% in water absorption.
- The water curing results in better mechanical properties compared to plastic curing.
- The use of *B. subtilis* in the microbial induced calcium carbonate precipitation (MICP) process was found to have the potential to self-heal cracks, as the healing process results in the filling of cracks with visible calcium carbonate minerals.
- The SEM analysis of the microstructure revealed a strong bond between the microcapsules and the mortar matrix, indicating the presence of calcium carbonate that was precipitated by *B. subtilis*.
- The EDS analysis of MICP showed the presence of increased bacterial growth due to the production of the enzyme urease, which contributes to the hydrolysis of urea and the subsequent precipitation of calcium carbonate as the dominant element.
- The use of *B. subtilis* demonstrated potential to enhance the mechanical properties and self-healing capabilities of mortar.

## Data Availability

The datasets used and/or analysed during the current study available from the corresponding author on reasonable request.
